# Characterization of *In Vitro* Resistance Development to the Novel Echinocandin CD101 in Candida Species

**DOI:** 10.1128/AAC.00620-16

**Published:** 2016-09-23

**Authors:** Jeffrey B. Locke, Amanda L. Almaguer, Douglas E. Zuill, Ken Bartizal

**Affiliations:** Cidara Therapeutics, Inc., San Diego, California, USA

## Abstract

CD101 is a novel echinocandin with a long half-life undergoing clinical development for treatment of candidemia/invasive candidiasis and vulvovaginal candidiasis. The potential for and mechanisms underlying the development of resistance to CD101 in Candida species were investigated by using spontaneous resistance and serial passage selection methodologies. Four Candida spp. (C. albicans, C. glabrata, C. parapsilosis, and C. krusei) were chosen for resistance characterization with CD101, anidulafungin, and caspofungin. The frequency of spontaneous, single-step mutations conferring reduced susceptibility to CD101 at 1× the agar growth inhibition concentration was low across all species, with median frequencies ranging from 1.35 × 10^−8^ to 3.86 × 10^−9^, similar to ranges generated for anidulafungin and caspofungin. Serial passage of Candida spp. on agar plates containing drug gradients demonstrated a low potential for resistance development, with passage 20 CD101-selected strains possessing increases in MICs equivalent to or lower than those for the majority of strains generated under selection with anidulafungin and caspofungin. A total of 12 *fks* “hot spot” mutations were identified, typically in strains with the highest MIC shifts. Cross-resistance was broadly observed among the 3 echinocandins evaluated, with no CD101-selected mutants (with or without *fks* hot spot mutations) exhibiting reduced susceptibility to CD101 but not also to anidulafungin and/or caspofungin. Consistent with currently approved echinocandins, CD101 demonstrates a low potential for resistance development, which could be further enhanced *in vivo* by the high maximum concentration of drug in serum (*C*_max_)/area under the concentration-time curve (AUC) plasma drug exposure achieved with once-weekly dosing of CD101.

## INTRODUCTION

Echinocandins target the 1,3-β-d-glucan synthase enzyme complex and mutations in the catalytic subunit-encoding *FKS* genes, which are associated with reduced susceptibility to echinocandins and increased clinical failure rates (1). Two specific “hot spots” within *FKS* genes (hot spot 1 [HS1] and HS2, encoding 9- and 8-amino-acid regions, respectively, of Fks1 and Fks2) are most often associated with reduced susceptibility to echinocandins (2). In C. glabrata, *FKS1* and *FKS2* are functionally redundant, and mutations can occur in either gene, whereas in other common Candida spp., such as C. albicans, C. tropicalis, C. parapsilosis, and C. krusei, only *FKS1* is essential, and mutations are limited to this gene (3). Although C. glabrata is not the most prevalent clinical Candida species, it is the most common species where echinocandin resistance is observed (4). This is due in part to its haploid state, and also, more recent work has identified a high prevalence of mutations in the DNA mismatch repair gene *MSH2* in clinical C. glabrata isolates, which can lead to rapid development of resistance to echinocandins as well as other antifungal drug classes (5).

CD101 is a novel echinocandin with antifungal potency and spectrum similar to those of the three currently approved echinocandins, caspofungin (CSF), micafungin (MCF), and anidulafungin (ANF) (6, 7), and is currently undergoing clinical development as an intravenous (i.v.) formulation for treatment and prevention of candidemia/invasive candidiasis and as a topical formulation for treatment of vulvovaginal candidiasis. Unlike currently approved echinocandins, CD101 can be administered intravenously once weekly and provides front-loaded, high-*C*_max_ (maximum concentration of drug in serum)/AUC (area under the concentration-time curve) plasma drug exposure due to its long human half-life (>80 h) and exceptional safety profile (8). Given that early therapeutic intervention/drug exposure is critical for the treatment fungal infections (9, 10), this front-loading dosing paradigm could be beneficial in improving efficacy over smaller daily doses (as has been observed with antibacterials) (11, 12) and could also have advantages in resistance prevention by maintaining drug concentrations in excess of the mutant prevention concentration (13).

Although echinocandins have been used clinically for the last 15 years, few studies characterizing the *in vitro* development of resistance to this drug class have been reported to date. In order to characterize the potential for and mechanisms underlying CD101 resistance development in Candida spp., both spontaneous mutation and serial passage methodologies were employed in this study, with CSF and ANF as comparators. Mutant strains were evaluated for cross-resistance trends and genetically characterized for the presence of *fks* hot spot mutations.

(Portions of this work were presented previously at the 55th Interscience Conference on Antimicrobial Agents and Chemotherapy, San Diego, CA, 17 to 21 September 2015 [posters F-753 and F-754] [34, 35].)

## MATERIALS AND METHODS

### Fungal strains and culture conditions.

Representative wild-type (WT) strains of C. albicans (NRRL Y-477; U.S. Department of Agriculture [USDA] Agricultural Research Service [ARS] culture collection), C. glabrata (ATCC 90030 and ATCC 2001; American Type Culture Collection), C. parapsilosis (CP02; J. Sobel, Wayne State University), and C. krusei (ATCC 6258) were chosen following prescreening on Sabouraud dextrose agar (SDA) plates containing each of the three echinocandins to ensure that they had clean, nonparadoxical growth phenotypes on agar media amenable for use with solid medium-based selection techniques.

### Antimicrobial agents.

Stocks of CD101 (formerly SP 3025, biafungin, AF-025; Seachaid Pharmaceuticals) were prepared fresh in 100% dimethyl sulfoxide (DMSO; Sigma) prior to use. The comparator antifungals ANF (Concord Chemicals Corporation), CSF (Molcan), and amphotericin B (AMB; Sigma) were also prepared in 100% DMSO according to Clinical and Laboratory Standards Institute (CLSI) guidelines (14).

### Antifungal susceptibility testing.

MIC assays were performed via broth microdilution in accordance with CLSI guidelines (14, 15), with the exception that test compounds were made up at a 50× final assay concentration and 100-μl assay mixture volumes were used (2 μl added to 98 μl of broth containing cells at 0.5 × 10^3^ to 2.5 × 10^3^ CFU/ml). All MIC assays were run at least three times, and a representative data set is shown. Quality control (QC) was assessed throughout the study via comparison of MIC values derived for WT C. krusei strain ATCC 6258 for AMB, CSF, and ANF with previously reported CLSI 24-h broth microdilution QC ranges (14).

### Selection of spontaneous mutants.

Large-plate-format, spontaneous, single-step resistance selection experiments were carried out as previously described, with appropriate modifications made for use with Candida species (16). Assay dishes (245 by 245 mm; Corning) were prepared with 150 ml SDA containing CD101, ANF, or CSF at the minimum concentration required to cleanly inhibit growth for each Candida strain. Three individual colonies from each strain were used to start cultures in RPMI. When cultures reached an optical density at 530 nm (OD_530_) of ∼1.0, they were pelleted and resuspended in 0.85% NaCl to a cell density of ∼1 × 10^8^ CFU/ml. One-milliliter aliquots were spread onto SDA plates containing the drug with sterile glass beads. The starting viable count was determined by triplicate plating of serial dilutions of the starting inoculum. Plates were incubated at 35°C for 48 h. Glycerol stocks of putative mutant colonies were stored at −80°C. Mutant resistance phenotypes were confirmed by subculturing on SDA plates containing an amount of drug equivalent to that used for initial selection. Spontaneous mutation frequencies were calculated by dividing the number of resistant colonies on a given plate by the starting inoculum plated. Mutant strains (including all of those selected with CD101 and a subset of those selected with ANF and CSF) were then evaluated by MIC and sequencing of *FKS* gene hot spot regions.

### Serial passage.

SDA drug gradient plates were created by pouring two overlapping layers of media as previously described (17). Briefly, the first layer containing 20 ml of drug-free SDA was poured into 90- by 90-mm-square petri dishes while on an incline. Once solidified, the plate was placed flat, and a second layer was poured, which contained CD101, ANF, or CSF at a concentration capable of fully inhibiting growth at a 1× concentration for each strain but allowing some growth past the edge of the plate containing no drug into the start of the drug gradient. Following each passage, the leading edge of growth (i.e., most resistant cells) was resuspended in 0.85% NaCl to an absorbance of an OD_530_ of ∼1.0, and a 100-μl aliquot (∼1.0 × 10^6^ CFU) was spread onto a fresh-passage plate using sterile glass beads. As strains developed reduced susceptibility to selecting drugs and were able to grow past the halfway point on the gradient plate, drug concentrations were increased 2-fold for subsequent passages. A glycerol stock was made from the total cell population under each culture condition for each passage. Twenty consecutive serial passages were completed for each drug-strain combination. MIC testing was performed on total cell populations for each group every fifth passage by using all selecting agents and AMB as a nonechinocandin control. Total population MIC data were plotted by using GraphPad Prism (GraphPad Software, Inc.).

### Analysis of individual passage 20 colonies.

Passage 20 (P20) total populations were streaked to isolation onto SDA, and three colonies were selected, capturing any colony size heterogeneity, if present. All three colonies were assessed via MIC, and a representative colony of the total population MIC was selected for further analysis.

### Sequence analysis of *FKS* gene hot spot regions.

Genomic DNA was isolated via the ZR Fungal/Bacterial DNA Miniprep kit (Zymo Research). Phusion Flash high-fidelity PCR master mix (Thermo Scientific) was used to amplify *FKS1* HS1 and HS2 regions as previously described (18). For C. glabrata strains, *FKS2* HS1 and HS2 regions were also amplified. PCR was performed by using an initial hot-start step at 98°C for 10 s followed by 30 cycles of 98°C for 5 s, 49°C for 5 s, and 72°C for 15 s and then a final 3-min extension step at 72°C. A DNA Clean and Concentrator-5 kit (Zymo Research) was then used to prepare samples for sequencing (Retrogen, Inc.) using upstream forward primers for each hot spot region. Mutations were identified by aligning mutant and WT *FKS* sequences using Vector NTI software (Thermo Fisher).

## RESULTS

### Determination of MIC values and spontaneous plating conditions.

The initial determination of broth MIC values informed the concentration ranges used to pour a series of test plates to determine the minimum concentration of each drug that would cleanly inhibit the growth of each Candida strain for the cell density plated. The final values chosen ranged from 1- to 4-fold higher than the corresponding CLSI broth MIC value for each strain (data not shown). Broth MIC values for AMB, CSF, and ANF derived for C. krusei ATCC 6258 in these preliminary assays as well as those generated in MIC assays following spontaneous and serial passage selection fell within recommended CLSI 24-h QC ranges (14). It should be noted, however, that CSF has inherently high interlaboratory MIC variability (19); therefore, the CSF MIC values reported here serve more reliably as a measure of relative fold shifts for mutants versus the WT parent strains than they do as stand-alone values.

### Spontaneous mutation frequencies.

A total of 472 spontaneous mutants were recovered following selection with CD101, ANF, and CSF versus 5 Candida strains (63 for CD101, 128 for ANF, and 281 for CSF) (Table 1). The spontaneous mutation frequencies for CD101 were low at 1× the agar plating inhibitory concentration, with median frequencies ranging from 1.35 × 10^−8^ to 3.86 × 10^−9^ across all 5 strains (Table 2). Median spontaneous frequencies for ANF and CSF were similar but demonstrated larger ranges (1.59 × 10^−7^ to <3.86 × 10^−9^ and 3.45 × 10^−7^ to <3.86 × 10^−9^, respectively). Both C. glabrata strains generated the highest number of mutants for all drugs overall. C. albicans mutation frequencies were lower than those for C. glabrata, with the exception of plating on ANF. C. parapsilosis and C. krusei had the lowest mutation frequencies, with the majority of plates generating 0 or 1 colonies for all three echinocandins (Table 1).

**TABLE 1:** Replicate assay mutant colony counts and spontaneous frequencies for CD101, anidulafungin, and caspofungin

Strain	Plate	CD101		ANF		CSF	
		No. of colonies	Frequency	No. of colonies	Frequency	No. of colonies	Frequency
C. albicans NRRL Y-477	1	4	5.00E−08	15	1.88E−07	1	1.25E−08
	2	2	2.27E−08	14	1.59E−07	1	1.14E−08
	3	7	5.47E−08	20	1.56E−07	1	7.81E−09
C. glabrata ATCC 90030	1	3	1.35E−08	2	9.01E−09	57	2.57E−07
	2	8	3.52E−08	1	4.41E−09	110	4.85E−07
	3	3	1.26E−08	5	2.10E−08	82	3.45E−07
C. glabrata ATCC 2001	1	9	3.16E−08	20	7.02E−08	9	3.16E−08
	2	13	3.79E−08	23	6.71E−08	10	2.92E−08
	3	3	1.17E−08	28	1.09E−07	9	3.50E−08
C. parapsilosis CP02	1	2	2.08E−08	0	<1.04E−08	0	<1.04E−08
	2	1	9.62E−09	0	<9.62E−09	0	<9.62E−09
	3	2	2.08E−08	0	<1.04E−08	0	<1.04E−08
C. krusei ATCC 6258	1	4	1.54E−08	0	<3.86E−09	0	<3.86E−09
	2	1	3.51E−09	0	<3.51E−09	1	3.51E−09
	3	1	3.86E−09	0	<3.86E−09	0	<3.86E−09

**TABLE 2:** Median spontaneous mutation frequencies for CD101, anidulafungin, and caspofungin

Strain	Median spontaneous mutation frequency		
	CD101	ANF	CSF
C. albicans NRRL Y-477	5.00E−08	1.59E−07	1.14E−08
C. glabrata ATCC 90030	1.35E−08	9.01E−09	3.45E−07
C. glabrata ATCC 2001	3.16E−08	7.02E−08	3.16E−08
C. parapsilosis CP02	2.08E−08	<1.04E−08	<1.04E−08
C. krusei ATCC 6258	3.86E−09	<3.86E−09	<3.86E−09

### Characterization of spontaneous mutants.

Of all the spontaneous mutants isolated, 73 were followed up with MIC and *FKS* hot spot sequence analyses (including all of those selected with CD101 and a subset of those selected with CSF and ANF, capturing any variability in MIC values, with a focus on less susceptible strains). Of these, 19 possessed *fks* mutations, resulting in 8 unique Fks substitutions, S645P, ΔF659, S663F, R665G, D666H, D666N, D666Y, and R1378S, across three strain backgrounds (C. albicans NRRL Y-477, C. glabrata ATCC 90030, and C. glabrata ATCC 2001) (Table 3). Because all CD101-selected mutants were sequenced, but only a subset of those selected with CSF and ANF were sequenced (focused on isolates with higher MIC values and representatives of each higher value, if available), CD101 is disproportionately represented as a selective agent in the strains presented in Table 3. Presumably because cross-resistance was observed between all mutants selected, CSF and ANF could have potentially selected mutations that happened to be present only in the CD101-selected mutants analyzed. The majority of the 63 spontaneous mutants selected with CD101 did not possess *fks* hot spot mutations, and *fks* mutants tended to have the largest MIC shifts (Table 4). Most mutants selected against C. albicans, C. parapsilosis, and C. krusei had insignificant increases in broth MIC values (≤2-fold) compared to those of the WT parent strains. C. glabrata mutants selected with all three echinocandins tended to have much higher fold MIC shifts that those of the parent strains, consistent with the presence of homozygous mutations inherent to the haploid state. All mutants demonstrating a ≥4-fold MIC shift for the selecting drug also demonstrated cross-resistance to at least one of the other echinocandins.

**TABLE 3:** Summary of MIC values for representative *fks* spontaneous mutants^*a*^

Background	Selecting drug(s)^*b*^	MIC (μg/ml)				Fks amino acid substitution			
		CD101	ANF	CSF	AMB	Fks1 HS1	Fks1 HS2	Fks2 HS1	Fks2 HS2
C. albicans NRRL Y-477	None	0.03	0.015	0.25	0.5	WT	WT	WT	WT
	CD101	0.25	0.25	0.5	0.25	S645P^*c*^	WT	NS	NS
C. glabrata ATCC 90030	None	0.06	0.06	0.25	0.5	WT	WT	WT	WT
	CD101, ANF	2	2	4	0.5	WT	WT	ΔF659	WT
	CD101, ANF	0.25	1	1	0.5	WT	WT	D666H	WT
C. glabrata ATCC 2001	None	0.125	0.06	0.25	0.5	WT	WT	WT	WT
	CD101, CAS	0.25	0.25	0.25	0.25	WT	WT	WT	R1378S
	CD101, ANF, CSF	2	4	16	0.25	WT	WT	ΔF659	WT
	CD101	0.5	0.5	0.5	0.5	WT	WT	D666Y	WT
	ANF	1	1	1	0.25	WT	WT	S663F	WT
	ANF	0.5	0.5	0.5	0.25	WT	WT	R665G	WT
	CD101	0.5	0.5	0.25	0.5	WT	WT	D666N	WT

a WT, wild type; NS, not sequenced.

b Of the 472 spontaneous mutants generated in spontaneous resistance experiments, all that were selected with CD101 were sequenced for the presence of *fks* mutations, whereas only a subset of those selected with ANF and CSF were sequenced.

c Heterozygous mutation.

**TABLE 4:** Susceptibility characteristics for CD101-selected spontaneous mutants with and without *fks* hot spot mutations

Strain	*fks* HS mutant			*FKS* HS WT		
	No. of colonies	Fold MIC shift		No. of colonies	Fold MIC shift	
		Median	Range		Median	Range
C. albicans NRRL Y-477	1	8	8	12	2	1–2
C. glabrata ATCC 90030	5	32	4–32	9	4	2–4
C. glabrata ATCC 2001	12	4	2–16	13	2	1–4
C. parapsilosis CP02	0	NA	NA	5	2	1–4
C. krusei ATCC 6258	0	NA	NA	6	1	1

### Analysis of MIC values of the total population after serial passage.

**FIG 1:**
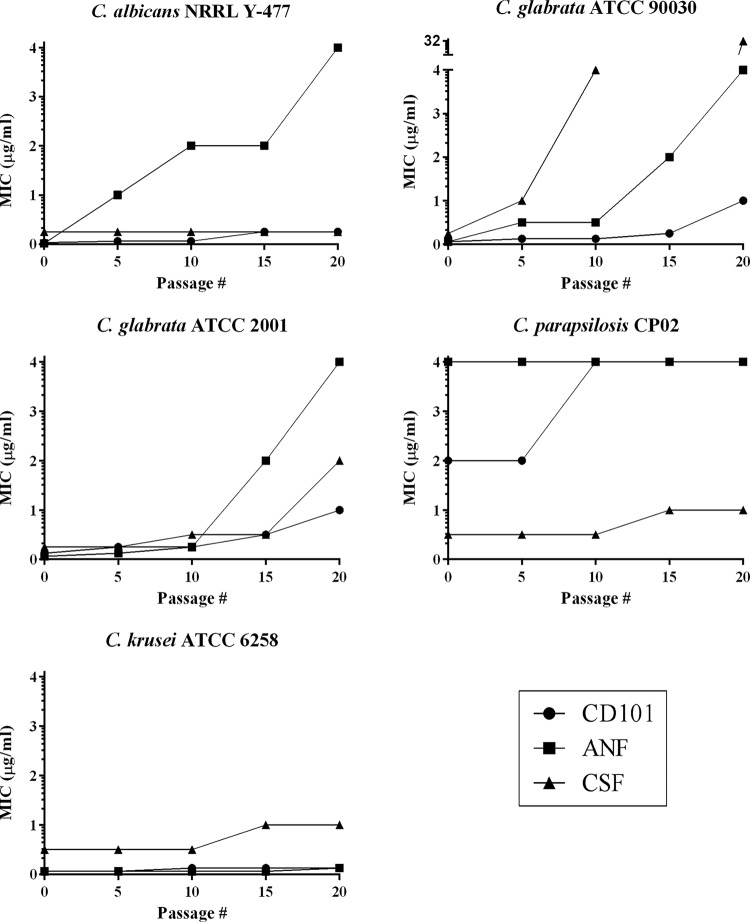
Gradient-plate serial passage MIC plots. MIC values for the selecting drugs CD101, anidulafungin (ANF), and caspofungin (CSF) were performed every fifth passage on total cell populations.

Drug solubility limitations for high concentrations of ANF and CSF in SDA medium for C. glabrata prevented serial passaging beyond P20. Every fifth passage, total population MIC values were assessed for each selecting drug and AMB versus strains from each of the drug/strain selection conditions. Following 20 passages, gradient-plate serial passage selection generated strains with high MICs of ANF (C. albicans and C. glabrata) and CSF (C. glabrata and C. krusei) (Fig. 1) (14), although again, it should be noted that MIC values for CSF should be viewed with caution when assessing susceptibility, as they are not as reliable as those for the other approved echinocandins (19). Because MIC breakpoints for CD101 have not yet been established, P20 CD101-selected strains could not be categorized as susceptible or resistant based on their MIC values. All three echinocandins demonstrated the largest MIC increases versus the two C. glabrata strains (Fig. 1). With the exception of ANF-selected C. albicans, all three drugs resulted in much lower fold MIC increases than the 3 non-C. glabrata species (Fig. 1). C. parapsilosis and C. krusei demonstrated the lowest potential for resistance development, with only ≤2-fold MIC shifts versus P20 strains selected with all three echinocandins.

### Analysis of individual serial passage 20 colonies.

All three single colonies analyzed from each of the P20 total populations had MIC values equivalent to the total population value for the selecting drug, and single representative colonies were assessed by MIC and underwent sequencing of *FKS* gene hot spot regions (Table 4). A total of 6 *fks* hot spot region mutations were identified, encoding Fks substitutions D632Y, S645Y, F659I, D666N, D666Y, and I1366S. With the exception of C. krusei, hot spot mutations were found only in P20 strains with the largest MIC shifts. The highest P20 MIC values for all three echinocandins occurred for the CSF-selected C. glabrata ATCC 90030 strain, resulting in the selection of two simultaneously occurring mutations (Fks2 F659I/D666Y) associated with a CSF MIC value of 32 μg/ml. Although all C. glabrata *fks* mutations are intrinsically homozygous, the C. albicans and C. krusei *fks* mutations identified in P20 colonies were also homozygous in these strains. No *fks* mutations were identified in P20 C. parapsilosis colonies, and this strain also had the lowest fold MIC shift increases of the 5 strains passaged. Cross-resistance was broadly observed across all strains and selecting drugs (Table 5). Strains selected with CD101 that had a 2-fold or greater CD101 MIC increase also had at least a 2-fold MIC increase for ANF and/or CSF.

**TABLE 5:** MIC values and Fks hot spot amino acid sequences of individual passage 20 colonies^*a*^

Background	Selecting drug	Strain^*b*^	MIC (μg/ml)				Fks hot spot amino acid sequence			
			CD101	ANF	CSF	AMB	Fks1 HS1	Fks1 HS2	Fks2 HS1	Fks2 HS2
C. albicans NRRL Y-477	None	WT	0.03	0.015	0.25	0.5	WT	WT	WT	WT
	CD101	P20-1^*c*^	0.25	0.125	0.5	0.5	WT	WT	NS	NS
	ANF	P20-1^*c*^	2	4	2	0.5	S645Y	WT	NS	NS
	CSF	P20-1	0.03	0.015	0.25	0.25	WT	WT	NS	NS
C. glabrata ATCC 90030	None	WT	0.06	0.06	0.25	0.5	WT	WT	WT	WT
	CD101	P20-2	1	1	1	0.5	WT	WT	WT	WT
	ANF	P20-1^*c*^	2	4	2	0.5	WT	WT	D666N	WT
	CSF	P20-1^*c*^	8	8	32	0.25	WT	WT	F659I, D666Y	WT
C. glabrata ATCC 2001	None	WT	0.125	0.06	0.25	0.5	WT	WT	WT	WT
	CD101	P20-2	1	1	1	0.25	WT	WT	WT	WT
	ANF	P20-2	2	4	2	1	D632Y	WT	WT	WT
	CSF	P20-2	2	2	2	0.5	WT	WT	WT	WT
C. parapsilosis CP02	None	WT	2	4	0.5	0.5	WT	WT	WT	WT
	CD101	P20-1	4	8	1	0.5	WT	WT	NS	NS
	ANF	P20-1	4	4	0.5	0.5	WT	WT	NS	NS
	CSF	P20-1	2	4	1	0.5	WT	WT	NS	NS
C. krusei ATCC 6258	None	WT	0.06	0.06	0.5	1	WT	WT	WT	WT
	CD101	P20-2	0.125	0.25	1	1	WT	I1366S	NS	NS
	ANF	P20-2	0.125	0.125	0.5	1	WT	WT	NS	NS
	CSF	P20-2	0.25	0.25	1	1	WT	I1366S	NS	NS

a WT, wild type; HS1, hot spot 1; HS2, hot spot 2; NS, not sequenced.

b Three colonies were analyzed from each passage 20 total population (i.e., P20-1, -2, and -3), and the colony chosen as a representative of each population is listed.

c Required 48 h of incubation to read MIC values.

## DISCUSSION

Through parallel spontaneous and serial passage resistance studies, we demonstrated that the potential for clinically relevant Candida spp. to develop reduced susceptibility to the novel echinocandin CD101 is low and similar to that for current members of the echinocandin class. Despite the value in characterization of *in vitro* resistance to help predict clinical resistance trends, very few studies assessing echinocandin resistance in Candida spp. through laboratory selection experiments have been reported (20–22). This work is the first comprehensive study to compare the resistance trends of multiple echinocandins across key Candida spp. using two different selection methodologies. These studies established baselines for other echinocandins and provided the opportunity to assess how the unique chemical structural features of CD101 may impact its potential for selection of resistance development.

The agar-based spontaneous mutation and serial passage methodologies employed in this study utilized empirically derived drug concentrations for each drug-strain combination. For echinocandins, this approach is more appropriate than simply basing agar drug concentrations on a multiple of the corresponding broth microdilution MIC values because echinocandin MIC values are read at 50% inhibition per CLSI guidelines, cell densities plated here are much higher than those used in CLSI broth microdilution MIC assays, and different inhibition dynamics can exist for cells plated on agar versus culture in broth media (15). This experimental paradigm is particularly important for CSF, as this drug has very high inherent interlaboratory variability in broth MIC values (19). Given this variability, the general resistance trends/frequencies derived for CSF here have much greater translatability than do the specific broth MIC values generated for CSF-selected mutant strains.

The frequencies of spontaneous, single-step mutations conferring reduced susceptibility to CD101 in the Candida strains tested were low. Mutants demonstrating reduced susceptibility to CD101 occurred at frequencies within ranges generated for ANF and CSF, and cross-resistance to ANF and CSF was broadly observed, suggesting a lack of mutations unique to selection with CD101. Previous studies involving the plating of C. albicans at higher fold multiples of the selecting echinocandin MIC value also generated low frequencies (i.e., ∼10^−8^) (21, 23). “Spontaneous mutant” colonies generated in this study grew on plates containing the selecting agent at 1× its inhibitory concentration on agar medium, and many of these strains demonstrated minimal changes in MIC values (i.e., ≤2-fold) that are not considered significant. This suggests that spontaneous mutations conferring >2-fold MIC shifts for all 3 echinocandins are even less frequent than those derived in these plating experiments. Phase 1 clinical trial pharmacokinetic data demonstrate that peak serum concentrations for CD101 following a single 400-mg dose could reach upwards of 23 μg/ml (8), providing a *C*_max_/MIC ratio well in excess of those for other echinocandins (and drug concentrations used in these spontaneous plating experiments), which could be beneficial in preventing resistance development and improving efficacy.

Through a perhaps more predictive measure of clinical resistance potential, Candida spp. were assessed via serial passage on CD101 drug gradient plates. This particular technique enables the selection of mutants across a broad range of drug concentrations from sub- to supra-MICs using a large number of cells, resulting in the rapid generation of reduced susceptibility compared to fixed-concentration liquid-based techniques, where resistance generation can occur much more slowly (20). In this format, CD101 performed well, suggesting that the potential for resistance development in Candida species is low, similarly to those for other echinocandins, with P20 fold MIC shift increases over the WT baseline being equivalent to or lower than those generated for 4 out of 5 strains for ANF and CSF. Future resistance studies employing additional strains would be helpful in determining if the trend toward less resistance development with CD101, particularly for C. glabrata, than with CSF and ANF will hold up. Cross-resistance was broadly observed among the three echinocandins evaluated, and there were no CD101-selected mutants (with or without *fks* hot spot mutations) that conferred reduced susceptibility to CD101 but not also to ANF and/or CSF. With the exception of C. krusei, only P20 strains with the largest MIC shifts possessed *fks* hot spot mutations. Consistent with clinical observations (4) and its haploid nature, C. glabrata demonstrated the highest potential for echinocandin resistance development.

Although selection through serial passage incorporates additional factors, such as fitness costs, compared to spontaneous, single-step selection, it would be expected that there would be a positive correlation between the two methods. In this study, the spontaneous resistance trends had a strong correlation with those observed for the same strain-drug combinations when assessed by serial passage. For example, C. parapsilosis and C. krusei had the lowest spontaneous mutation frequencies for all echinocandins, and they also had the smallest fold shifts in MIC values through serial passage. C. albicans had very low spontaneous mutation frequencies for CD101 and CSF but not for ANF, consistent with the serial passage observations. Finally, C. glabrata overall had the highest spontaneous mutation frequencies (in particular for CSF versus ATCC 90030), which held up in the parallel serial passage resistance trends.

With the exception of the Fks1 I1366 residue in P20 C. krusei, there is clinical precedence for *fks* mutations affecting each of the other 7 Fks hot spot residues for which mutants were selected in this study (2, 24), giving credence to the utility of *in vitro* resistance selection studies for echinocandins. Although there were common hot spot residues affected by *fks* mutations selected in both studies (C. albicans Fks1 S645 and C. glabrata Fks2 D666 and F659), C. glabrata Fks2 D666N and D666Y were the only two mutations generated in both the spontaneous and serial passage mutants analyzed. In clinical isolates, *fks* mutations affecting Fks1 S645 in C. albicans and Fks2 S663 and F659 in C. glabrata are some of the most commonly observed mutations and confer some of the largest MIC (and glucan synthase 50% inhibitory concentration [IC_50_]) shifts (25, 26), and all three of these residues were impacted by *fks* mutations selected in this study. Although *in vitro* echinocandin *fks* mutant data are limited, others have also identified mutations in C. albicans affecting Fks1 S645 (S645P/F/Y) though serial selection (21, 26). Although all non-C. parapsilosis spontaneous mutants with CD101 MIC values of >0.5 μg/ml possessed *fks* HS mutations, there were some C. glabrata P20 serial-passage strains with CD101 MIC values of 1 μg/ml without HS mutations. It would be informative to sequence the entire *FKS1* and *FKS2* genes in these strains to see if non-HS mutations, such as premature stop codons, could be contributing to their resistance phenotype as others have described previously (27).

One inherent constraint to the combination of echinocandins and Candida spp. in agar-based selection methodologies is that the strains used are limited to those not expressing a paradoxical phenotype on agar media. Fortunately, C. glabrata, the Candida species with the highest incidence of clinical echinocandin resistance (28), is also entirely nonparadoxical and is amenable to such resistance selection formats (29). Other clinically relevant species, such as C. albicans and C. tropicalis, have a much lower proportion of nonparadoxical members when plated onto agar media. Despite the screening of a large number of isolates, we were unable to identify a C. tropicalis representative that had a nonparadoxical phenotype strong enough to generate a clean, no-growth background on agar media (data not shown). The paradoxical nature of Candida, however, is lost in the presence of human serum (30) and has not been conclusively linked to *in vivo* virulence (31–33). Devising resistance selection methodologies amenable for use with paradoxical Candida strains as well as analysis of a greater number of strains for each species, including clinical isolates in addition to C. parapsilosis CP02, would help strengthen the confidence of any trends or comparative relationships observed in this study.

Despite the unique structural features of CD101, we have demonstrated that all CD101-selected mutants generated in this study were cross-resistant to other echinocandins. Because CD101 has *in vitro* resistance potential among Candida spp. similar to those of other echinocandins, yet is dosed clinically in a front-loaded manner that results in high plasma drug exposure, it is possible that CD101 could help reduce the potential for the emergence of resistance during therapy.
